# Brain Re-Irradiation Robustly Accounting for Previously Delivered Dose

**DOI:** 10.3390/cancers15153831

**Published:** 2023-07-28

**Authors:** Christopher Thompson, Christopher Pagett, John Lilley, Stina Svensson, Kjell Eriksson, Rasmus Bokrantz, Jakob Ödén, Michael Nix, Louise Murray, Ane Appelt

**Affiliations:** 1 Leeds Cancer Centre, Department of Medical Physics, Leeds Teaching Hospitals NHS Trust, Leeds LS9 7TF, UK; christopher.thompson5@nhs.net (C.T.);; 2 RaySearch Laboratories, SE-104 30 Stockholm, Sweden; 3 Leeds Cancer Centre, Department of Clinical Oncology, Leeds Teaching Hospitals NHS Trust, Leeds LS9 7TF, UK; 4 Leeds Institute of Medical Research at St James’s, University of Leeds, Leeds LS2 9JT, UK

**Keywords:** re-irradiation, treatment plan optimisation, robustness, equivalent dose

## Abstract

**Simple Summary:**

Repeat radiotherapy to a part of the body that has previously been treated with radiotherapy is challenging. It is difficult to determine where the dose previously went and how to safely deliver the best possible radiotherapy re-treatment. A tool designed to create better re-treatment plans is developed. In this work, we evaluate the safety of the new tools, which is an essential step before using it to make changes to patient treatments.

**Abstract:**

(1) Background: The STRIDeR (Support Tool for Re-Irradiation Decisions guided by Radiobiology) planning pathway aims to facilitate anatomically appropriate and radiobiologically meaningful re-irradiation (reRT). This work evaluated the STRIDeR pathway for robustness compared to a more conservative manual pathway. (2) Methods: For ten high-grade glioma reRT patient cases, uncertainties were applied and cumulative doses re-summed. Geometric uncertainties of 3, 6 and 9 mm were applied to the background dose, and LQ model robustness was tested using *α*/*β* variations (values 1, 2 and 5 Gy) and the linear quadratic linear (LQL) model δ variations (values 0.1 and 0.2). STRIDeR robust optimised plans, incorporating the geometric and *α*/*β* uncertainties during optimisation, were also generated. (3) Results: The STRIDeR and manual pathways both achieved clinically acceptable plans in 8/10 cases but with statistically significant improvements in the PTV D98% (*p <* 0.01) for STRIDeR. Geometric and LQ robustness tests showed comparable robustness within both pathways. STRIDeR plans generated to incorporate uncertainties during optimisation resulted in a superior plan robustness with a minimal impact on PTV dose benefits. (4) Conclusions: Our results indicate that STRIDeR pathway plans achieved a similar robustness to manual pathways with improved PTV doses. Geometric and LQ model uncertainties can be incorporated into the STRIDeR pathway to facilitate robust optimisation.

## 1. Introduction

Re-irradiation (reRT) is utilised in a number of tumour sites, usually with very limited levels of evidence [1,2,3,4,5,6]. Much of the data are heterogeneous, retrospective and likely subject to selection bias, resulting in conflicting reports of efficacy and toxicity. In addition, some of the heterogeneity in the existing data may relate to inconsistent approaches to the planning, evaluation and reporting of reRT [7].

In particular, there are almost no planning software tools for use specifically in the reRT setting [8]. Instead, a ‘post hoc’ approach to the evaluation of cumulative doses to organs at risk (OARs) is often used, based on maximum OAR doses in the two (or more) treatment plans: a typical approach might include extracting the point maximum OAR dose from the original irradiation (origRT) and the reRT treatment plans, manually converting the two values to an equivalent dose in 2 Gy fractions (EQD2) or biological equivalent dose (BED), and summing the two [9]. While this provides a simplified radiobiological representation of the combined dose, allowing the clinician to assess whether cumulative doses are acceptable, it disregards any spatial information (i.e., the location of the maximum dose in the origRT is unlikely to coincide with that in the reRT), nor does this approach work for any type of volumetric dose constraints, and it does not directly guide plan optimisation. In particular, while providing a ‘worst case’, this approach often overestimates and is limited to the cumulative maximum dose. Consequently, re-treatment target doses may be limited unnecessarily or placed in a suboptimal location [10]. The more refined approach of per-voxel biological dose summation is now becoming incorporated into clinical use for plan evaluation [11]. However, the use of this information for plan optimisation is currently ill defined.

A proposed improvement to such manual reRT workflows is the STRIDeR pathway (Support Tool for Re-Irradiation Decisions guided by Radiobiology), which is currently in development in collaboration with RaySearch using the RayStation TPS [12,13]. The tool has the capacity to optimise a reRT treatment plan using the origRT dose distribution as the background dose, after mapping the origRT dose to the reRT dataset. Uniquely, cumulative EQD2 objectives are then applied and optimisation in EQD2 is performed at a voxel-by-voxel level, thus overcoming the issues with manual EQD2 summation. Options to apply organ-specific repair and *α*/*β* values are also included within the STRIDeR pathway, which has previously been described in detail [13].

However, the robustness of reRT plans requires separate consideration, as modern radiotherapy delivers highly conformal doses to PTV targets, while sparing local OARs with steep dose gradients. Even when considering full dose distributions for assessment of cumulative doses, a cautious approach may be appropriate when optimising and evaluating reRT plans, as summed doses are subject to a number of additional uncertainties [11]. For example, there are often significant challenges in dose mapping from the origRT planning CT to the reRT planning CT, due to changes in patient anatomy over time resulting in imperfect image registration. This can partly be managed by the use of deformable instead of rigid registration, but there still are remaining uncertainties in the voxel-level dose mapping [14]. Additionally, the use of the linear quadratic model for calculation of equieffective doses to account for fraction-size effects (and possibly tissue recovery effects) represents another source of uncertainty [15,16]. Normal tissue *α*/*β* values are often not well defined. Further, the LQ model may not be accurate at higher doses per fraction [17,18], and adapted models have therefore been proposed.

The aim of the current study was to validate the robustness of the STRIDeR approach, taking uncertainties in cumulative OAR doses into account. We explored the use of the STRIDeR tool for ten patients treated for limited locally recurrent high-grade glioma. These patients experienced recurrence within or close to the previously irradiated high-dose region. reRT plans generated using the STRIDeR approach were compared to plans created using a more standard manual point maximum dose approach (subsequently referred to as ‘manual pathway plans’), in terms of plan quality as well as robustness to EQD2 model assumptions, alterations in *α*/*β* values, different image registration strategies, and errors in dose mapping. In addition, the impact of incorporation of geometric and *α*/*β* robustness during re-optimisation within the STRIDeR pathway was explored.

## 2. Materials and Methods

### 2.1. Patient Cases

Datasets for ten patients who had previously received reRT for recurrent high-grade glioma at Leeds teaching hospitals between 2008 and 2020 were used. Permission for use of the radiotherapy data within this project was provided from a Research Ethics Committee (REC) approved radiotherapy database management board (LeedsCAT, REC reference 19/YH/0300).

The original radiotherapy (origRT) prescription doses varied depending on the clinical scenario and included 54 Gy (*n* = 1), 55 Gy (*n* = 3) and 60 Gy (*n* = 5), all delivered in 30#. These plans were used as the background dose during optimisation of a reRT dose of 35 Gy in 10 fractions. Case-by-case variations in the position of origRT PTV and retreatment PTV, both in relation to each other and pertinent OARs, resulted in a variety of optimisation scenarios. However, all patients received re-irradiation type 1, as per the recent ESTRO-EORTC consensus statement [8], with overlap between the previous high dose and re-irradiation target. The reRT GTV consisted of areas of enhancing disease on T1 post-gadolinium MRI. An optional 5–10 mm CTV margin was added in selected cases at the clinician’s discretion, with manual edits at anatomical boundaries to spread, for example, bone. A 5 mm isotropic margin was added to the CTV to form the PTV. Clinical OAR contours were reviewed and edited as necessary by an experienced oncologist to ensure consistency. Planning Organ at Risk Volumes (PRV) margins were all 3 mm.

### 2.2. Treatment Planning Strategy and Cumulative Dose Evaluation

All plans used RayStation, 11A DTK (a non-clinical research-only release). Plans were for a VersaHD Elekta LINAC, using a 180-degree VMAT arc ipsilateral to the reRT PTV (re-RT PTV) with a second 45-degree cranio-caudal arc as previously described [19]. All beams used 6 MV FFF, while optimisation objectives for re-RT PTV, OAR and dose conformity were kept consistent between manual and STRIDeR plans. Dose was calculated on a 3 mm grid. Python scripts were used to ensure consistent plan settings and to access the EQD2 optimisation features (not yet implemented in the RayStation graphical user interface).

The planning priority was to meet OAR constraints (Table 1) while maximising re-RT PTV coverage, aiming to deliver the prescription dose to D50 where possible. Cumulative normal brain doses were additionally limited as far as possible to reduce the risk of radionecrosis [20]. Final plans were created by an experienced medical physicist and independently checked.

For all cases, plans produced using the STRIDeR pathway were compared to manual pathway plans (both described below), by evaluation of the clinical goals in Table 1. For evaluation of cumulative dose metrics, we mapped the origRT dose to the reRT CT, for summation with the reRT dose in EQD2. For per-voxel rescaling of 3D dose distributions to EQD2, we used the LQ model with *α*/*β* = 2 Gy and 25% recovery (for origRT) for all normal tissues.

(1) $$\mathrm{EQD}2=R\times D\left(\frac{d+\alpha /\beta \;}{2+\alpha /\beta \;}\right)$$

where *D* is the total dose, *d* is the dose per fraction and R is the recovery factor.

Baseline plan optimisations used *α*/*β* = 2 Gy and 25% recovery (i.e., 0.75 recovery factor for previously delivered dose prior to subtracting from the cumulative constraint) when taking the origRT background dose into account.

### 2.3. Image Registration

Original and reRT CT scans were registered either rigidly, using a correlation-based voxel intensity method, or deformably, using a hybrid intensity and structure-based method [22]. It was found that in regions of geometric change, particularly around the tumour bed, DIR performance was improved by the inclusion of ventricle contour matching as an additional constraint to the DIR algorithm.

To assess the impact of DIR vs. RIR on clinical goals, dose mapping to the reRT CT was performed using both registrations.

### 2.4. STRIDeR Planning Pathway

The STRIDeR EQD2 optimisation pathway has previously been described [13]. In brief, the STRIDeR pathway allows for the background dose to be used directly to account for the previously delivered dose in the plan optimisation.

The main pathway stages are

- (Deformable) image registration with organ-specific quality assessment;
- Mapping of origRT dose distribution to reRT dataset;
- Radiobiological optimisation in EQD2, voxel-by-voxel, using origRT dose as background dose;
- Dose summation in EQD2 for plan evaluation;
- Pathway options include number of fractions, *α*/*β* per OAR and recovery per OAR.

### 2.5. Manual Planning Pathway

For the manual pathway plans, a ‘dose remaining’ approach was used. Practically, the OAR PRV D_0.1cc_ previously delivered was converted to EQD2, subtracted from the relevant cumulative OAR EQD2 clinical goal from Table 1 to give the EQD2 remaining, which was then converted back to the retreatment fractionation. This ‘dose remaining’ OAR clinical goal was then applied to the retreatment physical plan optimisation objective. Effectively, this assumes that the entire organ received a uniform dose during the primary treatment, equivalent to the small volume maximum. To improve this approach for the brainstem, superior, mid and inferior thirds were used to improve geometric localisation of the previously delivered dose.

### 2.6. Robustness Evaluation (Re-Summed Plans)

For both STRIDeR and manual pathways, a series of robustness tests were used to examine how well the accumulated dose (based on deformable image registration) still fulfilled the clinical goals. The impacts of geometric, *α*/*β* value and LQ model uncertainties were examined.

To evaluate the impact of geometric uncertainty in image registration and in the resulting dose mapping to the reRT CT scan, origRT dose resampling was used. Our kernel resampled the background dose voxel-by-voxel and assigned the maximum dose sampled from the surrounding sphere of voxels within radius *r* (see Table 2 for test values). Dose summation was then performed using this new background maximum dose for both the STRIDeR and manual baseline plans without re-optimisation.

To assess *α*/*β* robustness in the STRIDeR and manual pathways, additional EQD2 summed doses were generated for plans originally optimised with *α*/*β* = 2 Gy and 25% recovery, using the same approach as described above (for the baseline plans) but using *α*/*β* values of 1 Gy and 5 Gy for dose summation, to reflect the range reported for cranial OAR [23,24].

Uncertainties related to the linear quadratic model used for all EQD2 calculations were examined using the Linear Quadratic Linear LQL model [18]

(2) $$\mathrm{EQD}2\left(LQL\right)=D\frac{\left(1+\frac{2d\;(\partial d-1+\exp\left(-\partial d\right)}{\frac{\alpha }{\beta }{\left(\partial d\right)}^{2}}\right)}{\left(1+\frac{2\partial -1+\exp\left(-2\partial \right)}{\frac{\alpha }{\beta }d^{2}}\right)}$$

where *D* is the total dose, *d* is the dose per fraction and δ is the term that determines the magnitude of the deviation from the LQ model. To assess LQ model robustness, the baseline plans were re-summed with a voxel-by-voxel application of Equation (2) for δ values 0.1 and 0.2, as reported [18].

### 2.7. Robust Optimisation

To demonstrate how uncertainties can be incorporated into the STRIDeR pathway, plans were additionally re-optimised to consider both geometric and *α*/*β* uncertainties during optimisation (rather than purely being recalculated using different geometric and *α*/*β* values, as above). Geometrically robust plans used the voxel-by-voxel resampled doses (described above at 3, 6 and 9 mm) as the background dose during optimisation, while for the *α*/*β* robust plans, duplicate optimisation objectives with *α*/*β* values 1 Gy and 5 Gy were used.

### 2.8. Data Analysis

**Figure 1:**
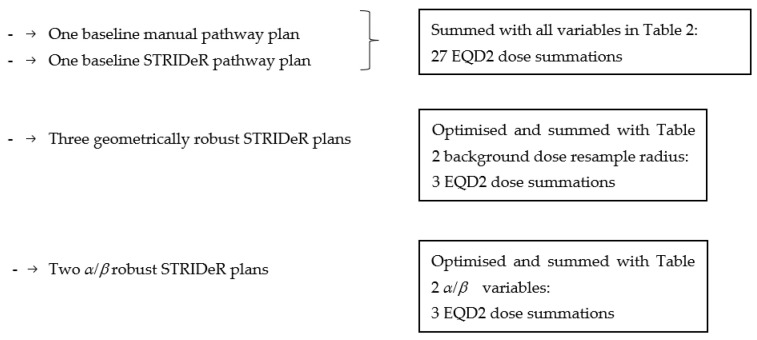
Overview of treatment plans created and dose summations performed for each patient case.

In summary, a total of 7 plans and 33 dose summations were created per patient (see Figure 1):

Wilcoxon signed-rank paired tests were used to compare both OAR-accumulated EQD2 doses and reRT plan PTV dose metrics for STRIDeR plans to manual pathway plans. *p* ≤ 0.05 was considered statistically significant (with no corrections for multiple testing).

A weighting approach for baseline plan comparison was also investigated to address concerns over dose values dominated by either previous or retreatment doses. This is discussed further in the Supplementary Material.

## 3. Results

### 3.1. Baseline Plans

For plans generated using the STRIDeR and manual pathways, cumulative OAR doses in EQD2 in Table 3 show marginally (but non-statistically significant) lower doses in the manual pathway. However, cumulative brain D0.1cc for one of the manual pathway plans exceeded 100 Gy EQD2, the threshold for an increased risk of radionecrosis [20]. This did not occur in any of the plans generated using the STRIDeR approach.

**Figure 2:**
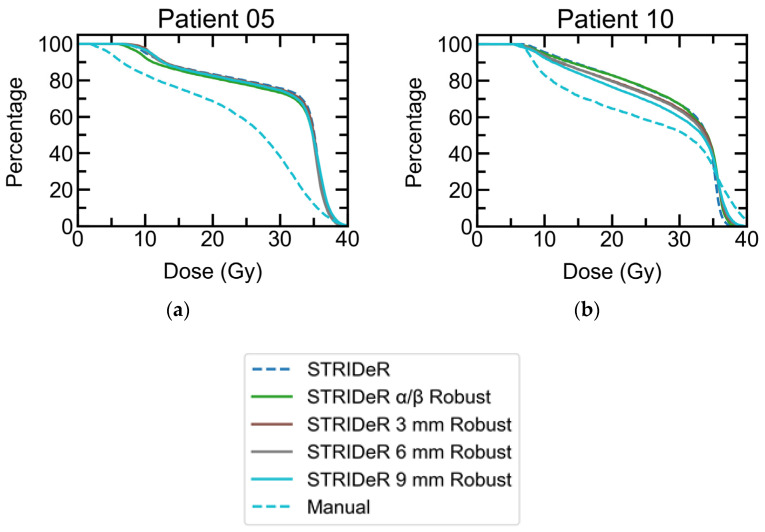
ReRT PTV DVH curves for STRIDeR and manual pathway baseline plans (dashed) and STRIDeR robust plans (solid) for (**a**) patient 5 and (**b**) patient 10, which showed the largest increase in OAR clinical goal failures when robustness was tested. Both patients required a significant PTV compromise to achieve clinical goals; a compromise that was markedly lower for STRIDeR compared to the manual pathway plans. The PTV coverage benefits in the STRIDeR plans were maintained when re-optimised to achieve robustness to the geometric and *α*/*β* uncertainty applied (thus removing clinical goal failures). For the remaining cases, all plots were overlaid, with good PTV coverage, and so are not presented.

Small but statistically significant differences were noted for PTV D98% (see Table 3) when comparing the baseline plans using the STRIDeR and manual approaches. PTV DVH curves (Figure 2) demonstrate the differences of greatest magnitude, which relate to cases 5 and 10. These two cases were unique in our small cohort in having a pronounced overlap between the origRT PTV (and thus high background dose) and the retreatment PTV, which was in close vicinity to the brainstem. In clinical practice, these two plans would have required a lower prescription and/or increased PTV compromise for the reRT plans to reach clinically acceptable brainstem doses. However, we used these relatively uncompromised plans to evaluate robustness, with maximum conflict between OAR dose and PTV coverage.

**Figure 3:**
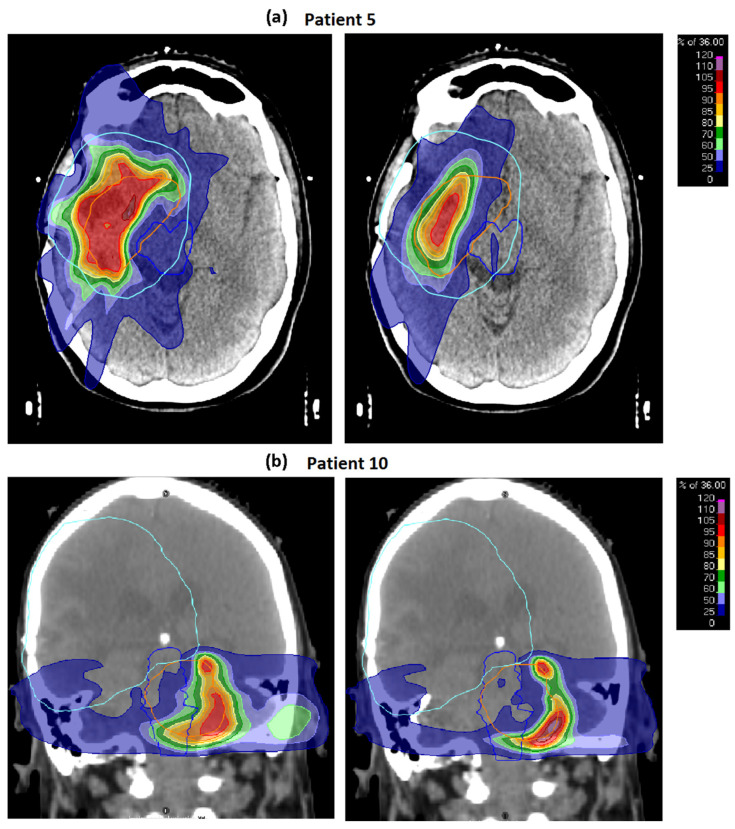
(**a**) Patient 5 and (**b**) patient 10, both with STRIDeR approach (left) and manual approach (right). ReRT PTV contour represented by orange outline, previous RT PTV contour represented by light blue outline, brainstem contour represented by royal blue outline. Greater compromise is observed in the manual plan as a result of the maximum dose received by the brainstem PRV being used to determine the dose remaining for the brainstem superior, mid and inferior divisions. In contrast, in the STRIDeR approach, the voxel-by-voxel EQD2 optimisation allows for appropriate local placement of the dose in the brainstem, resulting in improved PTV coverage. (For STRIDeR vs. manual; Case 5: PTV D95% 10.4 Gy vs. 5.2 Gy, PTV D80% 25.3 Gy vs. 12.6 Gy; Case 10: PTV D95% 10.8 Gy vs. 7.7 Gy, PTV D80% 22.8 Gy vs. 11.4 Gy.)

Figure 3 shows dose distributions for cases 5 and 10 with PTV compromises adjacent to the brainstem to meet the cumulative EQD2 clinical goal. In both cases, STRIDeR EQD2 optimised plans had higher PTV D95%/D98%, compared to the manual pathway plans, as a result of the voxel-by-voxel EQD2 optimisation achievable within the STRIDeR approach (in contrast to the manual approach where each of the three brainstem subdivisions (sup/mid/inf) used a uniquely calculated maximum dose).

The number of OARs exceeding their cumulative EQD2 clinical goal and the magnitude by which the goal was exceeded are reported in Table 4 for the baseline plans. The STRIDeR and manual method exceeded the OAR clinical goal on one occasion each by up to 0.2 Gy. These failures occurred in the most challenging cases (i.e., cases 5 and 10), despite the planning priority to meet OAR constraints.

There were no statistically significant differences in clinical goals evaluated using rigid versus deformable registrations for either the STRIDeR or the manual pathways.

### 3.2. Robustness

#### 3.2.1. Geometric Robustness

As expected, for re-summed plans, increasing the background dose sample radius correlated with additional failures in the cumulative dose clinical goals. Comparing the STRIDeR and manual pathway plans (Table 4), organs where cumulative constraints were exceeded and the resultant cumulative OAR dose above the constraint were marginally higher in STRIDeR—i.e., the geometric robustness of STRIDeR plans was potentially lower.

STRIDeR plans optimised with the resampled origRT dose as the background dose (resampling as described for the geometric robustness optimisation testing) resulted in more robust plans with fewer cases of OAR constraints being exceeded, with only a minor impact on PTV coverage (Figure 2). Failures still occurred for these patients; as background doses exceeded OAR constraints in 0, 1 and 2 cases based on a resampling radius of 3 mm, 6 mm and 9 mm, respectively, meaning there was no practical way to reduce cumulative doses below constraints.

Geometrically robust optimisation within STRIDeR resulted in maintained PTV coverage with reduced episodes of constraint violation. This indicates that the maximum dose approach for optimisation of the brainstem dose in the manual pathway, where fewer episodes of constraint violation occurred, was likely over-conservative, resulting in a lower PTV coverage than potentially achievable.

#### 3.2.2. Fractionation Sensitivity

Changes to the *α*/*β* value, applied at the dose summation stage, were also associated with a number of cases exceeding the OAR dose constraints. The magnitude of the dose excess above the constraint was less than that observed for the geometric analysis, with STRIDeR and the manual pathway of equal robustness. Unlike the geometric robustness, where dose gradients caused the largest change in summed dose, *α*/*β* impacted across areas of uniform backgrounds and retreatment doses such that OAR failures did not correlate with those observed with geometric testing.

For the *α*/*β* robust optimised STRIDeR plans, there was only one case of a dose constraint being exceeded across the *α*/*β* range used (Table 4).

#### 3.2.3. LQ Model Robustness

Summation of the doses with the LQL model applied (as opposed to the LQ model) had minimal impact on the STRIDeR and manual plan clinical goal failures, resulting in a small increase in total dose exceeding the constraints only (Table 4). The only failure of note was patient 5 (optic nerve), which also failed during *α*/*β* robust summation. Manual pathway plans had a higher excess dose (above constraint).

### 3.3. Weighting Factor

Our results when weighting based on the proportion of dose attributable to the re-irradiation (Supplementary Material Figure S1) were identical to those presented here.

## 4. Discussion

We have demonstrated that the use of the STRIDeR approach is feasible for retreatment planning of high-grade glioma patients with recurrent disease. When compared to the manual approach, OAR doses were equivalent, while PTV coverage was improved. In cases where high doses overlapped adjacent to a critical OAR, PTV coverage was most improved but still compromised to meet organ constraints. This planning advantage is a result of increased geometric information and the voxel-by-voxel approach utilised during STRIDeR plan optimisation, allowing optimised plans to sculpt the dose based on full background dose information. In our cohort, only 2/10 cases displayed a high dose overlap of the PTVs in critical OAR(s), and thus the significant improvement in PTV coverage was mainly driven by this subset of patients. Further benefits might be seen if a higher proportion of such patients are selected for re-irradiation.

Previous work has demonstrated the application of the STRIDeR pathway to manage reRT planning, with selective, OAR-specific use of deformable image registration for dose mapping to allow the use of the previous dose distribution as the background dose to guide radiobiological optimisation of a re-irradiation plan [13]. Where the deformable image registration was considered unreliable, a ‘dose remaining’ approach was adopted but, again, with consideration of radiobiology. In this current work, we explore the uncertainties associated with the STRIDeR pathway, including incorporating dose mapping uncertainties into dose evaluation and plan optimisation.

Acceptance of deformable registration for routine clinical use is a challenge [25], though the potential benefit is increasingly recognised in the reRT setting [26,27], though robust approaches are recommended [14,28]. For this cohort of brain patients, however, our results indicate that there was no significant difference when reverting to a more accepted rigid registration, likely because the anatomical change in this cohort was fairly minimal between treatment courses. The impact of different registration processes may be greater in other anatomical sites. Our previous work [13] explores this issue in more detail and provides an alternative pathway for OARs where image registration is unreliable. However, our results clearly indicate that image registration uncertainties can be robustly integrated into the plan optimisation process, without any degradation of plan quality.

Intuitively, the manual planning approach might be assumed to result in a more robust plan when geometric uncertainties are incorporated into the cumulative dose evaluation. This follows from the fact that this process applies the maximum dose originally received across the whole OAR (or portion of OAR in the case of the brainstem), which is potentially over-conservative. However, this was not conclusively borne out in our data, where STRIDeR plans were generally as robust as manual plans for geometric dose mapping uncertainties. The one exception may be instances where the OAR is adjacent to a high dose gradient: for one case (patient 5), the STRIDeR plans were less robust in terms of OAR doses but with improved PTV coverage (based on re-summed plans). This could be corrected for the STRIDeR plans by incorporating geometric uncertainties into the background dose used at optimisation.

The approach of resampling the background dose within an ellipsoid has previously been suggested when evaluating summated doses and robustness of these in the re-irradiation setting [29]. Our novel use of this approach during EQD2 plan optimisation generated plans with greater geometric robustness than a manual approach, with improved PTV coverage (Figure 2). In our work we simplified the analysis by selecting a uniform, isotropic kernel for the entire patient. However, there is no reason to limit this approach and an ovoid kernel could be used with either per voxel or per organ differentiation.

Uncertainties in *α*/*β* values were also investigated and were shown to have the potential to cause summed doses exceeding applied OAR constraints, even in organs where geometric robustness was adequate. For the range of *α*/*β* values sampled, uncertainties could be mitigated in the STRIDeR robust optimised plans by including a series of objectives with differing *α*/*β* values with a minor impact on the PTV doses achieved.

LQL model testing indicated that the dose per fraction was sufficient in these plans to have a small impact on the summated doses. A maximum change of 4.0 Gy was observed for these patients when using δ = 0.2. For plans with a very high dose per fraction, therefore, it may be appropriate to generate summed doses using alternative models, and then, if necessary, re-optimise to reduce doses to OARs below acceptable limits.

In summary, to reduce the risk of unintended dose hot spots within OARs, a robust optimisation approach for the STRIDeR pathway, as demonstrated here, is recommended. Robust planning when using a manual point maximum dose optimisation is likely to be iterative and result in a further compromise in PTV coverage in the absence of the geometric information required to guide the optimisation. The STRIDeR pathway offers a solution that can incorporate these robustness considerations directly into the plan optimisation.

The study was limited to ten patients and, given the varied nature of the re-irradiation scenarios, a larger study would be useful to add weight to our observations and allow more detailed statistical analyses of plan metrics, potentially with more cases where significant OAR cumulative doses occur. The impact of uncertainties in recovery is also not included. However, we have shown that by incorporating uncertainties during optimisation, the STRIDeR pathway can be used with greater confidence.

As discussed above, our use of a universal dose resampling kernel could be refined to a per-organ kernel and given dimensions appropriately estimated on a per organ basis. One could go further, estimating geometric uncertainty on a voxel-wise basis. For sites outside the head, this becomes more critical, as DIR uncertainties can be large and highly inhomogeneous. Quantification and localisation of such uncertainties are ongoing challenges in DIR but could be leveraged very effectively by our robust re-irradiation planning approach. While inhomogeneous, organ-specific kernels could be desirable in a clinical pathway, we considered that the approach adopted here simplified the plan comparison between the different pathways. Our *α*/*β* uncertainty approach follows a similar argument.

In addition to the reported results, while using the two workflows, it was observed that optimisation objectives working in EQD2 were less prone to the user errors seen in manual calculation. The improved STRIDeR workflow is further facilitated by the use of templates to load standard clinical goals and optimisation objectives, not possible when setting patient-specific values in the manual planning strategy. In RayStation, the planner is also able to review total EQD2 dose metrics (not 3D dose) during optimisation to confirm acceptable OAR doses. In the STRIDeR workflow, it is anticipated that clinician involvement can be mandated less frequently, resulting in a less iterative convergence on the final planning solution. A well-designed timing study is needed to quantify these advantages within a radiotherapy department environment and will be completed as part of future work as the RayStation GUI for reRT matures. A final additional anticipated advantage will be the clear electronic record of summed doses, which could inform future dose-toxicity modelling work.

## 5. Conclusions

The STRIDeR tool in development in RayStation has been used for glioma brain reRT treatment planning and assessed for robustness. The STRIDeR tool offers voxel-by-voxel cumulative EQD2 optimisation, thereby allowing appropriate dose sculpting in the reRT setting. Issues related to uncertainties in geometry, *α*/*β* values and the models used to convert one fractionation to another can impact on cumulative OAR doses and PTV coverage. These factors should therefore be considered when planning reRT. The STRIDeR reRT approach is one where such uncertainties can be incorporated.

## Figures and Tables

**Table 1:** Organ At Risk (OAR) clinical goals [19] in equivalent dose in 2 Gy per fraction (EQD2) and target physical dose re-RT metrics for plan evaluation. Planning Organ at Risk Volume (PRV) = OAR + 3 mm margin. PTV: Planning Target Volume. Summed doses were calculated with 25% repair applied to the origRT dose.

Structure	Metric	Cumulative Constraint EQD2
Brainstem PRV	D0.1cc (Gy)	51.3
	Dmean (Gy)	48.5
Orbit	D0.1cc (Gy)	39.4
Optic Chiasm PRV	D0.1cc (Gy)	51.3
Optic Nerves PRV	D0.1cc (Gy)	51.3
re-RT PTV	D95% (Gy)	
	D98% (Gy)	
	Dmean (Gy)	
PxDose Spillage [21]	=Vol(100%)/PTV V100%	
Modified gradient index [21]	=Vol(50%)/PTV V100%	

**Table 2:** Summary of the robustness parameters tested.

		Values		
Robustness Test	Variable	Min	Mid	Max
Geometric	Sampling Radius, r (mm)	3	6	9
Fraction Sensitivity	*α*/*β* (Gy)	1	2	5
LQL	δ	0	0.1	0.2

**Table 3:** Population mean and inter-quartile range (IQR) cumulative organ at risk (OAR) doses, in equivalent dose in 2 Gy fractions (EQD2) for the baseline STRIDeR and manual pathways. Planning target volume (PTV) metrics are presented in physical dose. The impacts of deformable and rigid registrations in cumulative OAR doses (in EQD2) are also presented. (DIR, deformable image registration; RIR, rigid image registration; IQR interquartile range).

Structure	Metric	STRIDeR Pathway Median (IQR)		Manual Pathway Median (IQR)		In-Patient Difference	Wilcoxon Signed Rank
		DIR Doses	DIR-RIR Doses	DIR Doses	DIR-RIR Doses	Strider-Manual Median (IQR)	*p*-Value
Brainstem PRV3	D_0.1cc_ (Gy)	44.5 (36.0−49.3)	0.0 (−0.9–0.3)	44.1 (34.1–47.4)	0.0 (−0.7–0.1)	0.7 (0.0–2.4)	0.05
	D_mean_ (Gy)	16.2 (9.5–33.0)	0.0 (−0.2–0.4)	16.7 (9.4–31.1)	0.0 (−0.1–0.4)	0.1 (0.0–0.6)	0.08
Left Eye	D_0.1cc_ (Gy)	3.0 (1.8–5.7)	0.0 (0.0–0.0)	2.6 (1.8–5.4)	0.0 (0.0–0.0)	0.1 (0.0–0.2)	0.14
Right Eye	D_0.1cc_ (Gy)	5.2 (1.7–15.5)	0.0 (−0.3–0.0)	5.4 (1.7–11.7)	0.0 (−0.3–0.0)	0.0 (0.0–0.3)	0.08
Optic Chiasm PRV3	D_0.1cc_ (Gy)	39.4 (17.9–45.5)	0.0 (−0.2–0.3)	39.4 (17.9–44.9)	0.0 (−0.2–0.1)	0.0 (−0.2–0.1)	0.62
Left Optic Nerve PRV3	D_0.1cc_ (Gy)	27.4 (3.4–38.3)	0.1 (0.0–0.2)	27.4 (3.3–37.8)	0.1 (0.0–0.1)	0.0 (−0.1–0.1)	0.46
Right Optic Nerve PRV3	D_0.1cc_ (Gy)	19.2 (7.2–40.7)	0.0 (−0.1–0.0)	19.7 (5.7–40.6)	0.0 (0.0–0.0)	0.0 (0.0–0.1)	0.28
Brain-PTV	D_mean_ (Gy)	24.0 (22.7–25.5)	0.0 (−0.1–0.1)	24.1 (22.6–25.7)	0.0 (−0.1–0.1)	0.0 (−0.1–0.2)	0.46
	D_10%_ (Gy)	51.2 (48.4–55.0)	−0.1 (−0.3−0.1)	50.5 (48.1–52.4)	−0.1 (−0.4−0.1)	0.0 (−0.2–0.2)	0.42
PTV PTV PTV	D_95%_ (Gy)	34.9 (34.6–34.9)	NA	34.9 (34.2–34.9)	NA	0.1 (0.0–0.4)	0.12
	D_98%_ (Gy)	34.5 (33.2–34.6)	NA	34.4 (32.0–34.5)	NA	0.1 (0.0–0.9)	**0.01**
	D_mean_ (Gy)	35.5 (35.5–35.5)	NA	35.5 (35.5–35.5)	NA	0.0 (0.0–0.1)	0.12
PxDose Spillage [21]	Vol(100%)/VPTV(100%)	1.11 (1.07–1.15)	NA	1.09 (1.06–1.11)	NA	0.0 (0.0–0.0)	0.22
Modified gradient index [21]	Vol(50%)/Vol(PTV)	3.5 (3.2–4.8)	NA	3.6 (3.3–5.1)	NA	−0.1 (−0.2–0.0)	0.88

Significance tests compare the in-patient differences using Wilcoxon signed rank, with statistically significant results marked in bold.

**Table 4:** Failure to meet individual OAR dose constraints and excess dose to OARs for STRIDeR and manual approaches for variations in *α*/*β* value and geometric robustness (maximum failures = goals × patients: 10 for brainstem and optic chiasm, 20 for optic nerves and eyes). ** The results for the robust optimised plans, i.e., geometric robust, used a resampled background dose during optimisation, while *α*/*β* robust plans used the two objective functions with *α*/*β* = 1 and *α*/*β* = 5 Gy per OAR.

Plan Type	Test Parameters	Number of Clinical Goal Failures				Total Dose Exceeding Constraints (Gy)
		Brain Stem	Optic Nerves	Optic Chiasm	Eyes	
**Baseline Plans**						
Manual pathway	*α*/*β* = 2 Gy Sample Radius = 0 mm	0	0	1	0	0.2
STRIDeR	*α*/*β* = 2 Gy Sample Radius = 0 mm	1	0	0	0	0.1
**Geometric Robustness Test**						
Manual (re-summation)	Sample Radius = 3 mm	0	0	1	0	1.5
	Sample Radius = 6 mm	0	0	1	0	2.3
	Sample Radius = 9 mm	3	0	1	1	8.9
STRIDeR (re-summation)	Sample Radius = 3 mm	2	0	1	0	2.6
	Sample Radius = 6 mm	2	0	1	0	5.6
	Sample Radius = 9 mm	4	0	1	2	21.6
STRIDeR robust (optimisation **)	Sample Radius = 3 mm	0	0	0	0	0.0
	Sample Radius = 6 mm	1	0	0	0	0.3
	Sample Radius = 9 mm	2	0	0	0	3.9
**LQ Model Robustness Test**						
Manual (re-summation)	*α*/*β* = 1 Gy	0	0	1	0	0.9
	*α*/*β* = 5 Gy	1	1	1	0	5.7
	δ = 0.1 (LQL model)	0	1	1	0	2.7
	δ = 0.2 (LQL model)	1	0	1	0	2.9
STRIDeR (re-summation)	*α*/*β* = 1 Gy	1	0	0	0	0.9
	*α*/*β* = 5 Gy	2	1	0	0	3.3
	δ = 0.1 (LQL model)	1	0	0	0	0.4
	δ = 0.2 (LQL model)	1	1	0	0	0.8
STRIDeR robust (optimisation **)	*α*/*β* = 1 Gy	0	0	0	0	0.0
	*α*/*β* = 2 Gy	0	0	0	0	0.0
	*α*/*β* = 5 Gy	1	0	0	0	0.6

## Data Availability

Non-patient data used in this study are available on request from the author.

## Supplementary Materials

The following supporting information can be downloaded at https://www.mdpi.com/article/10.3390/cancers15153831/s1, Figure S1: Weighting Factor.
