# Supplementary material for: Brain Re-Irradiation Robustly Accounting for Previously Delivered Dose
Source: Cancers (Basel). 2023 Jul 28;15(15):3831. doi: 10.3390/cancers15153831 (PMC10417278; doi:10.3390/cancers15153831)
Supplement: Supplementary file 1 [file cancers-15-03831-s001.zip › cancers-2486319-supplementary.pdf]

Supplementary Materials:.

S1. Weighting factor

A comparison was made to address concerns over dose values dominated by either previous or retreatment dose masking significance in retreatment planning results.

Plan quality was assessed using DVH statistics reported by RayStation. A significant difference in a DVH statistic may be hidden when taking averages/ median values for multiple plans. For example when the background dose is high, the impact of a change in planning technique on the total dose is limited (as the main contribution to the total dose comes from the origRT. Conversely as the dose from the background plan reduces towards zero the plan becomes dependent on current re-irradiation doses only, such that the significance of any differences is less indicative of a technique advantage.

For all plans, and all DVH statistics, therefore, the ratio of previous dose to re-irradiation dose was calculated between the reRT plan and the summed plan dose. A weighted mean was then calculated for the given statistic using a weighting factor with a maximum at 0.5 for the ratio of previous to re-irradiation dose figure.S1.

The weighting approach discussed here had no significant impact on results reported.

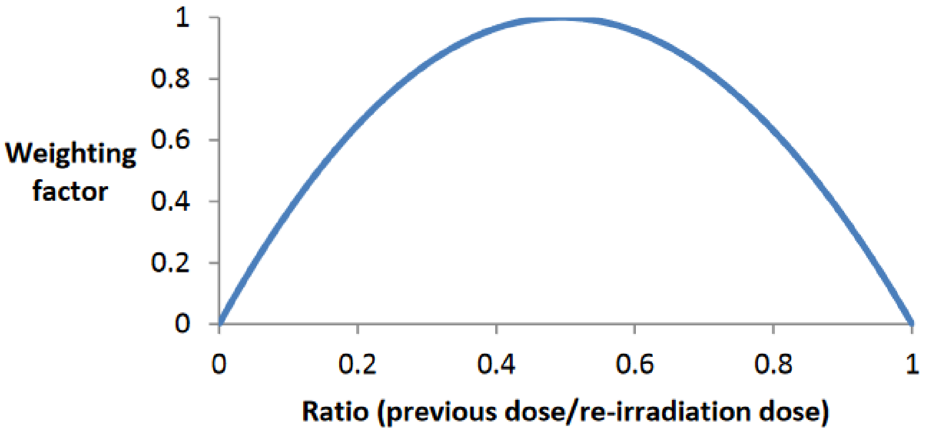

Figure S1. DVH Statistic Weighting Factors.
